# Beyond the naked eye: a systematic review on the current state of radiomics approaches to the vestibular schwannoma

**DOI:** 10.1007/s11060-026-05663-8

**Published:** 2026-06-16

**Authors:** Rithvik Gundlapalli, Purushotham Ramanathan, Veda Akula, Douglas Fox, Matthew Nguyen, Derek Meyers, Xin He, Mariam Ishaque, Ryan T. Kellogg, Benjamin D. Lovin, Jason Sheehan, Adam Thompson-Harvey, Georgios Maragkos, Ashok Asthagiri

**Affiliations:** 1 University of Virginia School of Medicine, Charlottesville, VA 22903 USA; 2 University of Virginia College of Arts and Sciences, Charlottesville, VA 22903 USA; 3 Department of Neurosurgery, University of Virginia, Charlottesville, VA 22903 USA; 4 Department of Otolaryngology - Otology, Neurotology & Skull Base Surgery, University of Virginia, Charlottesville, VA 22903 USA

**Keywords:** Vestibular schwannoma, Radiomics, Machine learning, MRI segmentation, Predictive modeling

## Abstract

**Purpose:**

Vestibular schwannomas (VS) present a clinical challenge in management decision-making due to their difficult-to-access location, unpredictable growth, and potential impact on crucial neurological function. This systematic review evaluates and summarizes the potential for radiomics, a computational tool that extracts quantitative features from imaging, to predict VS clinical outcomes and assess treatment responsiveness.

**Methods:**

Studies were extracted by searching PubMed, OVID Medline, and Web of Science databases. Included studies analyzed radiomic features from MRI as independent variables and varied in their methodology to predict clinical outcomes. Studies evaluated associations between radiomic features, pre-procedural clinical features, and post-procedural outcomes.

**Results:**

Thirteen retrospective studies met inclusion criteria; eleven of these used machine learning models to analyze radiomic MRI features. One non-ML study correlated longitudinal tumor volumetric changes with texture features. All segmentation workflows utilized manual or semi-automated approaches to determine the lesion’s region of interest. Models based on pre-procedural imaging demonstrated moderate predictive accuracy by Area Under the Receiver Operating Characteristic curve (AUC = 0.66–0.7), while post-procedural models showed moderate to strong predictive capacity (AUC = 0.75-1.0). One study employed a convolutional neural network evaluating postoperative facial nerve outcomes (AUC = 0.89) that outperformed traditional ML models (AUC = 0.64–0.85).

**Conclusion:**

Radiomics-based predictive modeling in VS shows encouraging preliminary results across a range of clinical outcomes. However, small sample sizes, retrospective designs, and lack of standardization and external validation in models hinder its widespread applicability. Addressing these limitations through prospective studies with standardized datasets and models, potentially incorporating deep learning, will be essential to improve generalizability and support clinical integration.

**Supplementary Information:**

The online version contains supplementary material available at 10.1007/s11060-026-05663-8.

## Introduction

Vestibular schwannomas (VS), historically also known as acoustic neuromas, are benign tumors arising from the Schwann cells insulating the vestibulocochlear nerve [1]. VS can manifest with a spectrum of hearing loss, tinnitus, facial nerve weakness, and balance dysfunction. Treatment options, depending on radiologic and clinical factors, include observation with serial imaging, microsurgical resection, or stereotactic radiosurgery (SRS). Management is complicated by the tumor’s size, extension into the internal auditory canal, brainstem compression, and proximity to critical neurovascular structures [2, 3], which are all incorporated into guidelines established by the Congress of Neurological Surgeons (CNS) and European Association of Neurological Oncology (EANO) [4, 5]. Many low-grade VS are initially observed for growth with serial imaging, given the risks of procedural intervention [4, 5]; however, the natural history of these tumors during this observation period is heterogeneous.

Radiomics, paired with machine learning (ML), offers a solution by predicting clinically relevant outcomes, such as growth, treatment response, and functional outcomes, to reduce clinical uncertainty, cost, and complications. Radiomics involves extracting quantitative, reproducible data from medical images beyond human perception [6]. As an analogy, in cancer genomics, a tumor is analyzed by isolating DNA and sequencing genes to assess for clinical correlation. Similarly, radiomics uses computer-based algorithms to quantitatively analyze medical imaging, such as MRI. These algorithms decompose the region of interest (ROI) into constituent parts, referred to as feature classes, including first-order grayscale intensity, second-order texture, and higher-order transform-based features. Just as genomics employs *genes* as predictive tools in the status of tumors, radiomics employs *features* in an analogous capacity.

Generating a radiomics profile for a given VS involves delineating the tumor boundaries in 3D space on MRI to produce a segmented ROI, followed by feature extraction and analysis. The most relevant features are selected through statistical analysis or ML models to predict outcomes such as tumor growth, treatment response, and hearing loss to ultimately inform management.

As a reference, ML refers to algorithms that learn patterns from data to make predictions or decisions, with methods such as random forest (RF) or support vector machines (SVMs). Deep learning (DL), a specialized branch of ML, uses multilayered artificial neural networks to model complex nonlinear relationships through hierarchical feature extraction without the need for predefined feature extraction [7].

Given the clinical need for radiomic predictive tools to serve VS patients and the ever-increasing growth of DL technology capable of interacting with radiomic data, this paper summarizes the current state of radiomics methodologies and poses future directions to predict outcomes in VS.

## Methods

### Literature search

A systematic review was conducted in accordance with the Preferred Reporting Items for Systematic Reviews and Meta-Analyses (PRISMA 2020) guidelines. A comprehensive literature search was performed across PubMed, OVID Medline, and Web of Science databases to identify studies investigating the relationship between radiomics features and a broad range of vestibular schwannoma radiographic and clinical outcomes from 01/01/2010 to 10/31/2024. The complete keyword search strings used per database are included as Supplement 1 to this manuscript. This review protocol was not prospectively preregistered.

### Study selection

All studies were screened by three independent reviewers (R.G./V.A./D.F.). Studies were eligible for inclusion if they extracted and assessed radiomic features from publicly available or local imaging sets with respect to clinical outcome variables in patients with VS. Clinical outcomes studied include pre-procedural clinical features and post-procedural outcomes.

Studies that did not have an English language version, worked with non-human subjects, assessed non-sporadic vestibular schwannomas, or did not assess radiomics in relation to clinical features or outcomes were excluded. Review articles, editorials, and case reports, were also excluded.

### Data collection

Several baseline characteristics were gathered from each study, such as design, methodology, and outcomes. Notably, only the radiomic features selected by statistical methods to be included in a predictive ML model or for final analysis were included. In studies with over 10 radiomic features represented, the feature classes were collected instead, along with a numerical count of the total features.

Studies were classified by the endpoints they evaluated. Pre-procedural clinical features assessed outcomes before any intervention, including hearing loss, VS blood supply, and tumor growth prediction. Post-procedural VS outcomes included response to SRS, transient tumor enlargement of VS after SRS, and postoperative facial nerve function.

Most studies trained an ML model to utilize radiomic feature data to predict clinical outcome, using a training dataset to build the model and a testing dataset to generate outcome parameters such as accuracy, validity, and area under the curve (AUC) of the receiver operating characteristic curve to evaluate the model. AUC data, when available, were tabulated to demonstrate the potential efficacy of such models in using radiomics to predict clinical outcomes.

### Risk of bias

A PROBAST Risk of Bias Assessment was done (Table 1) to evaluate the risk of bias in each of the included studies. Of the 13 studies evaluated, 7 were found to be at an overall high risk of bias, with the remaining 6 bearing some cause for concern. The most common source of bias was found in analysis, with no studies conducting any external validation and the majority having risk of overfitting bias and/or reporting bias. Complete data for PROBAST is included as Supplement 2. Qualitative synthesis of the studies was done, but quantitative meta-analysis was not practical given significant heterogeneity between study methodologies. This systematic review protocol was not prospectively preregistered, which represents a methodological limitation and may increase risk of reporting bias.

**Table 1:**
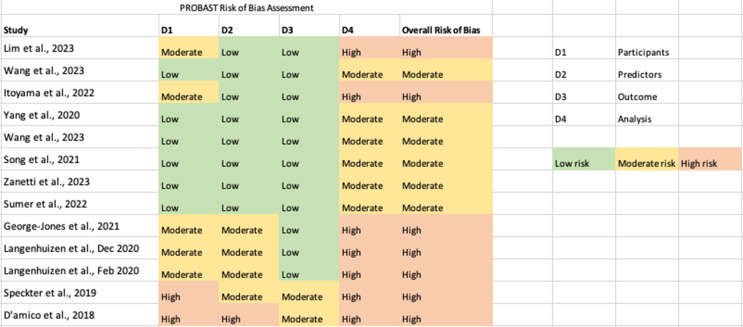
PROBAST risk of bias assessment for the studies included in this systematic review

## Results

### Overview of studies

**Fig. 1:**
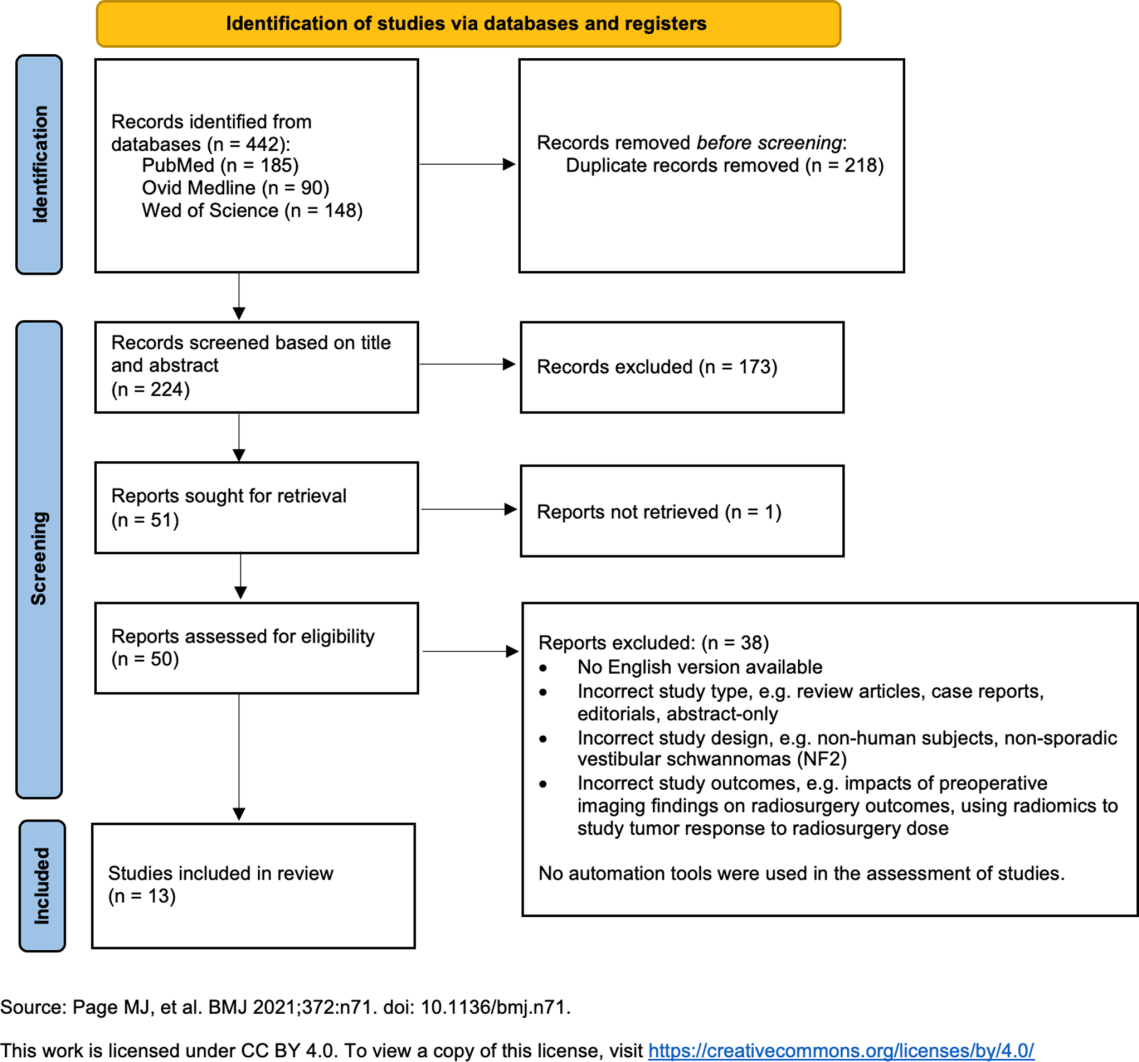
Identification of articles for qualitative synthesis based on PRISMA search guidelines

Thirteen studies met the eligibility criteria (Fig. 1**)**, all of which employed a retrospective design [8–20]. Four studies assessed pre-procedural clinical features in VS (Table 2), and nine evaluated post-procedural outcomes. Twelve of thirteen studies employed ML models in handling radiomic data to predict clinical outcomes and utilized radiomic features from patient index imaging to build predictive models. Three studies employing radiomics also incorporated clinical data (e.g., age, comorbidities) into their predictive algorithms. Seven studies evaluated radiomics related to SRS outcomes, four to natural history (growth or baseline hearing), and two to surgical outcomes.

The studies evaluating radiomic feature relevance to clinical VS outcomes demonstrated significant heterogeneity in their methodology (Tables 2 and 3). Tumor segmentations were done either manually or semi-automatically, with the most popular software being Slicer3D (Slicer.org), MRIcro (www.cabiatl.com/mricro), VelocityAI (Varian Medical Systems, Palo Alto, CA), ITKSnap (Version 3.8.0, PICSL, USA), and GammaPlan (Elekta AB, Stockholm, Sweden). Radiomic feature extraction was obtained through a combination of internal and external (Pyradiomics, Slicer3D). ML models included SVM, RF, and neural networks.

Given the heterogeneity of studies, a meta-analysis was not performed; instead, results were synthesized qualitatively.

**Table 2:**
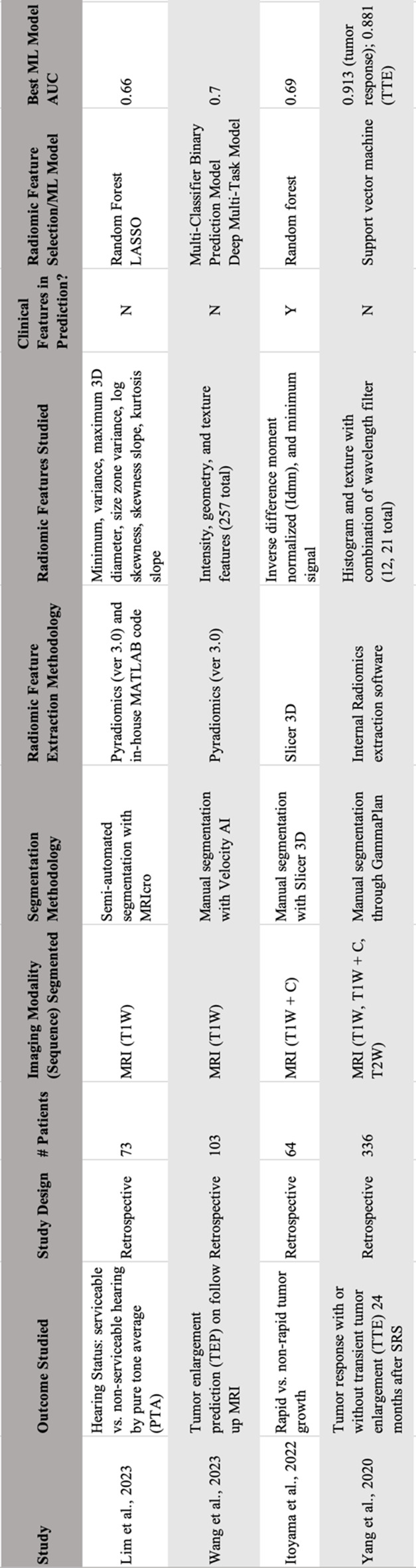
Overview of studies utilizing radiomic features in determination of pre-procedural clinical Outcomes in VS. Abbreviations: PTA = pure tone average; TEP = tumor enlargement prediction; LASSO = least absolute shrinkage and selection operator; MRI = magnetic resonance imaging; T1W = T1-weighted; T1W + C = T1-weighted with contrast

**Table 3:**
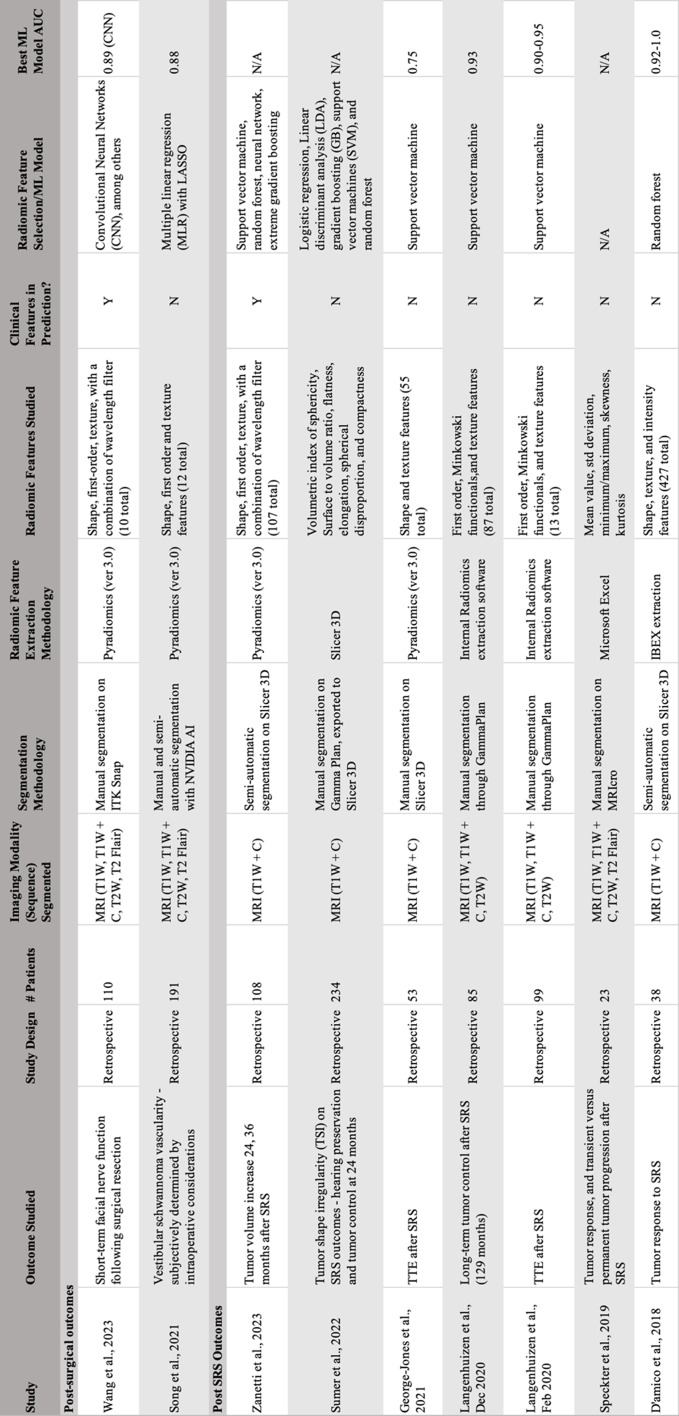
Overview of studies utilizing radiomic features in determination of post-procedural clinical outcomes in VS. Abbreviations: T1W = T1-weighted; + C = with contrast; T2W = T2-weighted; TTE = transient tumor enlargement; SRS = stereotactic radiosurgery; IBEX = Imaging Biomarker Explorer; CNN = convolutional neural network; MLR = multiple linear regression; LASSO = least absolute shrinkage and selection operator; LDA = linear discriminant analysis; GB = gradient boosting; SVM = support vector machines

## Clinical outcomes

### Pre-procedural clinical features

Propensity for growth is a critical feature for VS prediction due to its association with neurologic deterioration, and is directly tied to management [4, 5]. Of the four studies evaluating pre-procedural clinical features in VS (Table 2), two evaluated radiomics in the setting of tumor growth prediction. Wang et al. (2023) compared a radiomics-based model with a deep multi-task model to evaluate tumor growth prediction, both found to have moderate efficacy with AUCs of 0.70 [21].

Similarly, Itoyama et al. (2022) found that a combined radiomics and clinical data ML model incorporating texture features and clinical variables had an AUC of 0.69 in predicting rapid VS growth, which outperformed pure clinical and pure radiomics models [22]. They quantified rapid growth as greater than 2 mm/year and found that both tumor heterogeneity and presence of cystic components, quantified by radiomic texture features, are predictors of growth [22]. Both Wang and Itoyama demonstrated moderate predictive performance for tumor growth [21, 22].

Yang et al. (2020) developed two ML models using preradiosurgical clinical predictors and MRI radiomic features to predict long-term tumor control (AUC 0.91) and to distinguish transient tumor enlargement (TTE) from true progression (AUC 0.88), demonstrating strong predictive performance in both clinical scenarios [13].

Evaluating hearing loss, Lim et al. used radiomic data against the clinical outcome of baseline serviceable versus non-serviceable hearing loss, based on air conduction pure tone average and word recognition scores, to produce a model with moderate predictive capacity (AUC of 0.66) [23]. While limited, Lim et al. demonstrates that MRI textures may be associated with hearing impairment.

### Post-procedural clinical outcomes

Seven of nine studies that evaluated post-procedural outcomes studied TTE, a temporary increase in tumor size occurring after SRS, measured as a percentage of tumor growth over time. They underscored the potential utility of radiomics in predicting TTE after SRS based on patients’ index imaging (Table 3); however, conclusions are limited by methodological heterogeneity and lack of external validation. Zanetti et al. utilized a neural network model that combined radiomic and clinical features, and the remaining seven studies evaluated radiomic features alone [8].

Speckter et al. (2019) was the only study not to utilize an ML model with radiomic data, but instead trended radiomic features and statistically correlated them to tumor enlargement across those same time points. They found that some texture features, such as T2-weighted minimum intensity, statistically correlated with final tumor reduction [14]. Zanetti et al. showed that the traditional ML models (RF and SVM) were insufficient compared to their neural network model in identifying patients with increased tumor volume.

The remaining two studies assessed surgical outcomes (Table 3). Wang et al. (2023) evaluated House-Brackmann scores to measure the severity of facial nerve dysfunction, using radiomic features from index scans. Using convolutional neural networks (CNN) with radiomic and tumor features (e.g., tumor size, CSF cap, remnant/recurrence), they produced a model with an AUC of 0.89 in predicting postoperative facial nerve outcomes [24]. Song et al. studied the outcome of tumor vascularity, which was retrospectively grouped into high or low vascularity based on intra-operative records, and developed a radiomic ML model with strong predictive capability(AUC 0.88) [25].

## Discussion

ML tools are being developed to support complex analyses of medical imaging, and their application to radiomic predictive modeling of VS outcomes is an area of ongoing exploration. These thirteen retrospective studies suggest moderate to strong results in correlating radiomics with valuable clinical factors and predictions in VS. Radiomics models have potential to be used in clinical practice, but need to be proven to be robust, generalizable, and clinically meaningful. External validation and prospective research are still significantly lacking. This review aims to illustrate how these studies present radiomic modeling as a potential resource for neurooncologists and neurosurgeons, outlining areas for future investigation into its role in informing clinical care of patients with VS.

### Radiomic prediction of hearing loss

Ipsilateral hearing loss is a critical feature of VS, and hearing preservation is a major goal in management [2]. EANO guidelines suggest observation in small, asymptomatic VS and support consideration for SRS when VS affect hearing function. Therefore, predicting which tumors are more likely to impact hearing based on radiomic features has potential to improve patient outcomes [5]. Lim et al. produced a model with some predictive capacity to identify baseline hearing loss when delineating between serviceable and non-serviceable hearing loss [23]. This model examined radiomic features associated with current, not future, hearing loss, demonstrating that radiomic data is associated with hearing loss. Still, consideration of more powerful models using DL to develop a true predictive model is needed, and future models might incorporate longitudinal imaging data or advanced algorithms to predict eventual hearing trajectory, rather than cross-sectional status. Early prediction can alter timing of intervention, patient counseling, or selection between observation, SRS, and surgery.

### Postoperative facial nerve function

Alongside hearing, preservation of facial nerve function is an essential goal in managing VS. Up to two-thirds of patients undergoing surgical resection of VS can have unsatisfactory House-Brackmann scores [16]. Therefore, Wang et al. (2023) built a CNN model incorporating radiomic and clinical data to predict short-term postoperative facial nerve function with strong predictive potential [24] based on facial nerve, measured by heterogeneity within an ROI, interpreted with first-order or high-order radiomic features [24]. Predicting facial nerve function can help determine intraoperative decision-making, nerve monitoring, and patient selection for surgical approaches.

### Intra-operative tumor vascularity

VS vascularity is a critical preoperative consideration as it can not only complicate the surgical resection, but can also affect preoperative decision making such as the need for preoperative embolization [17, 18]. Tumor vascularity could also impact the effect of ionizing radiation on the underlying tumor. Song et al. created a radiomics model that correlated with intraoperative records of tumor vascularity [25]. While this model showed strong predictive performance (AUC 0.88), the impact of utilizing subjective intraoperative records introduced uncertainty. Despite this, the ability of radiomics-based models to predict the extent of tumor vascularity and the potential for DL to augment this process is promising and warrants further investigation. Future studies emphasizing angiographic correlates of vascularity are likely to be beneficial, especially to better determine preoperative embolization decisions and surgical risk counseling.

### Transient tumor enlargement and tumor response after SRS

Much of the literature on radiomic modeling in VS outcomes focuses on tumor response to SRS, which can include stable disease, TTE, partial response, and complete resolution of tumor. Identifying tumors likely to respond to SRS is vital for selecting the appropriate treatment. SRS can lead to TTE, where radiation causes a transient increase in tumor volume due to connective tissue buildup [13], which is particularly dangerous in larger VS where TTE may exacerbate mass effect [12]. Langenhuizen et al. identified no consistent clinical factors associated with TTE, making differentiation between TTE and true tumor progression difficult [12]. Historically, only the time interval could help distinguish between TTE and true progression, with incidence of TTE peaking at 6–18 months post-SRS and true progression at 2 years [13].

Radiomic modeling shows encouraging preliminary results in predicting tumor response and TTE following SRS. Zanetti et al. produced a neural network ML model that satisfactorily predicted tumor response at 24 and 36 months post-SRS, though there was decreasing sensitivity and specificity over time [8]. Langenhuizen et al. also had promising results with a radiomics model assessing tumor response at 24 months for tumors larger than 5 cm^3^, suggesting that larger tumors may be better candidates for radiomics evaluation for post-SRS outcomes [11].

George-Jones et al. used radiomic modeling at a median time of 6.5 months post-SRS to detect enlargement, while later studies by Yang et al. and Langenhuizen et al. aimed to distinguish TTE from true tumor growth [10, 12, 13]. Yang et al. created a two-level ML model with high accuracy in distinguishing between TTE and tumor progression. Overall, the literature supports radiomic modeling in assessing response to SRS in favor of TTE and tumor response at 24 months, particularly for larger tumors greater than 5 cm^3^.

Other notable studies assessing SRS response include the work by Sumer et al., which utilized radiomic modeling to characterize tumor shape irregularity, which was not found to correlate with clinical outcomes such as tumor control at 2 years [9].

### Applications, limitations, and future directions

Radiomics models reviewed in this paper can be utilized in clinical practice by selecting an outcome of interest, extracting radiomics features, and inputting them into a given study’s ML model to provide clinically relevant results, which can be applied to patient care within the ML model’s limitations. However, in practice, implementation of these workflows is extremely challenging, as many ML radiomics pipelines require labor-intensive manual or semi-automated segmentation of imaging data, often on a pixel-by-pixel basis. Reproducing such workflows across large clinical volumes would require substantial time and expertise, representing a deeply rooted practical limitation of many traditional ML-based radiomics approaches.

While powerful, ML models still face limitations in their methodology. Concerns remain in generalizability of ML models used in medicine [19], as image acquisition, segmentation workflows, ROI delineation, and feature extraction and analysis vary significantly and impact the reproducibility of radiomic features [20, 25]. Due to high heterogeneity in study methodologies and lack of open-source tools, interstudy generalizability is low. For example, features correlated with TTE in one study may not demonstrate similar associations in another. Heterogeneity also makes generalizability low between topics. For example, extracting radiomic features for tumor growth prediction in one study would require redoing the entire process for a different outcome, such as response to SRS.

The reliance on manual preprocessing and feature selection distinguishes many traditional ML radiomics workflows from emerging DL and unsupervised learning approaches, which may offer more automated workflows that reduce user burden. While ML models have their limitations, these thirteen studies demonstrate that there is possibility within the realm of radiomic data to drive clinically-relevant predictions; the next frontier for radiomics-based analysis lies in the development of DL models like CNNs that prioritize multi-institutional datasets, standardized preprocessing, and external validation across independent cohorts to ensure that predictive models maintain performance across diverse clinical settings. The use of consensus frameworks such as the Image Biomarker Standardization Initiative (IBSI) [26, 27] may be key.

An additional consideration for future radiomics development is the architectural design of predictive models. One strategy is the creation of a unified “super-model” capable of predicting multiple clinically relevant outcomes, such as tumor growth, hearing preservation, and treatment response, within a single framework. Such models may benefit from the large, diverse datasets and the integration of multimodal inputs. However, unified models may introduce challenges related to model complexity and interpretability, whereas task-specific modeling strategies, where parallel models are trained to address individual clinical questions, may allow for more targeted optimization and easier validation across specific outcomes.

To fully maximize the potential of the ever-evolving radiomics and ML technologies in creating predictive models, the development of standardized workflows across different stages of the radiomics pipeline is essential [28]. Initiatives like the Vestibular-Schwannoma-SEG Database, with standardized instructions for image pre-processing, lesion segmentation, and radiomics extraction, are in their infancy, but collaboration across large medical image datasets with standardized processing would enable the creation of more robust ML models with higher fidelity than isolated studies [29].

## Conclusion

Radiomics-based predictive modeling for clinical outcomes in VS patients shows encouraging potential based on emerging literature investigating the prediction of pre- and post-procedural clinical parameters and outcomes. However, current progress is limited by several barriers, including small sample sizes, reliance on retrospective data, variability in imaging protocols, and lack of external validation in predictive modeling approaches, making cross-study comparison difficult. Standardized large-scale image databases with multicenter, prospective, and external validation are essential for future model development to create more robust DL models and improve outcome generalizability.

## Supplementary Information

Below is the link to the electronic supplementary material.

Supplementary Material 1

Supplementary Material 2

## Data Availability

No datasets were generated or analysed during the current study.

## Abbreviations

AUC: Area under the curve
CNS: Congress of neurological surgeons
CNN: Convolutional neural network
DL: Deep learning
EANO: European association of neurological oncology
ML: Machine learning
PRISMA: Preferred reporting items for systematic reviews and meta-analyses
RF: Random forest
ROI: Region of interest
ROC: Receiver operating characteristic
SRS: Stereotactic radiosurgery
SVM: Support vector machine
TTE: Transient tumor enlargement
VS: Vestibular schwannoma
