# Supplementary material for: Beyond the naked eye: a systematic review on the current state of radiomics approaches to the vestibular schwannoma
Source: J Neurooncol. 2026 Jun 16;178(3):67. doi: 10.1007/s11060-026-05663-8 (PMC13272267; doi:10.1007/s11060-026-05663-8)
Supplement: Supplementary file 1 — Supplementary Material 1 [file 11060_2026_5663_MOESM1_ESM.docx]

**Beyond the Naked Eye: A Systematic Review on the Current State of Radiomics Approaches to the Vestibular Schwannoma**

Search: PubMed, Ovid Medline, Web of Science

Search Date: 01/01/2010 to 10/31/2024

Search Terms:

**PubMed:**

(Acoustic Neuroma OR Acoustic Neuromas OR Acoustic Schwannoma OR Acoustic Schwannomas OR Acoustic Neurinoma OR Acoustic Neurinomas OR Acoustic Neurilemoma OR Acoustic Neurilemomas Neurilemomas OR Vestibular Schwannoma OR Vestibular Schwannomas) AND (radiomics OR Histogram features OR Gray level histogram OR gray level mean OR gray level maximum OR gray level minimum OR gray level variance OR gray level percentiles OR Gray level skewness OR gray level kurtosis OR Texture features OR Absolute gradient OR Gray Level Cooccurrence Matrix OR Gray Level Run-length Matrix OR Gray Level Size Zone Matrix OR Gray-Level Distance Zone Matrix OR Gray Tone Difference Matrix OR Neighborhood Gray Tone Difference Matrix OR Gray Level Dependence Matrix OR Neighborhood Gray Level Dependence Matrix OR Model based features OR Transform-based methods OR Fourier wavelet transforms OR Gabor wavelet transforms OR Haar wavelet transforms OR Shape based features OR Compactness OR Sphericity OR Density) [185 results]

**OVID Medline:**

(Acoustic Neuroma OR Acoustic Neuromas OR Acoustic Schwannoma OR Acoustic Schwannomas OR Acoustic Neurinoma OR Acoustic Neurinomas OR Acoustic Neurilemoma OR Acoustic Neurilemomas Neurilemomas OR Vestibular Schwannoma OR Vestibular Schwannomas) AND (radiomics OR Histogram features OR Gray level histogram OR gray level mean OR gray level maximum OR gray level minimum OR gray level variance OR gray level percentiles OR Gray level skewness OR gray level kurtosis OR Texture features OR Absolute gradient OR Gray Level Cooccurrence Matrix OR Gray Level Run-length Matrix OR Gray Level Size Zone Matrix OR Gray-Level Distance Zone Matrix OR Gray Tone Difference Matrix OR Neighborhood Gray Tone Difference Matrix OR Gray Level Dependence Matrix OR Neighborhood Gray Level Dependence Matrix OR Model based features OR Transform-based methods OR Fourier wavelet transforms OR Gabor wavelet transforms OR Haar wavelet transforms OR Shape based features OR Compactness OR Sphericity OR Density) [90 results]

**Web of Science:**

(Acoustic Neuroma OR Acoustic Neuromas OR Acoustic Schwannoma OR Acoustic Schwannomas OR Acoustic Neurinoma OR Acoustic Neurinomas OR Acoustic Neurilemoma OR Acoustic Neurilemomas Neurilemomas OR Vestibular Schwannoma OR Vestibular Schwannomas) AND (radiomics OR Histogram features OR Gray level histogram OR gray level mean OR gray level maximum OR gray level minimum OR gray level variance OR gray level percentiles OR Gray level skewness OR gray level kurtosis OR Texture features OR Absolute gradient OR Gray Level Cooccurrence Matrix OR Gray Level Run-length Matrix OR Gray Level Size Zone Matrix OR Gray-Level Distance Zone Matrix OR Gray Tone Difference Matrix OR Neighborhood Gray Tone Difference Matrix OR Gray Level Dependence Matrix OR Neighborhood Gray Level Dependence Matrix OR Model based features OR Transform-based methods OR Fourier wavelet transforms OR Gabor wavelet transforms OR Haar wavelet transforms OR Shape based features OR Compactness OR Sphericity OR Density) [148 results]

Search yielded 442 studies, 218 of which were manually screened out as duplicates.

224 studies were screened for eligibility, and 13 studies were found to meet the inclusion criteria.

**Inclusion/Exclusion Criteria:**

Inclusion criteria: Extracted and assessed radiomics features with or without the use of an ML model; Analyzed clinical outcome variable in patients with vestibular schwannoma

Exclusion criteria: no English version; non-human subjects; non-sporadic vestibular schwannoma; abstracts/editorials; background/review articles; response/commentary articles; study design or outcome data not specified or not exclusive to radiomics and radiographic or clinical outcomes for vestibular schwannoma
