# Supplementary material for: Beyond the naked eye: a systematic review on the current state of radiomics approaches to the vestibular schwannoma
Source: J Neurooncol. 2026 Jun 16;178(3):67. doi: 10.1007/s11060-026-05663-8 (PMC13272267; doi:10.1007/s11060-026-05663-8)

**Beyond the Naked Eye: A Systematic Review on the Current State of Radiomics Approaches to the Vestibular Schwannoma**


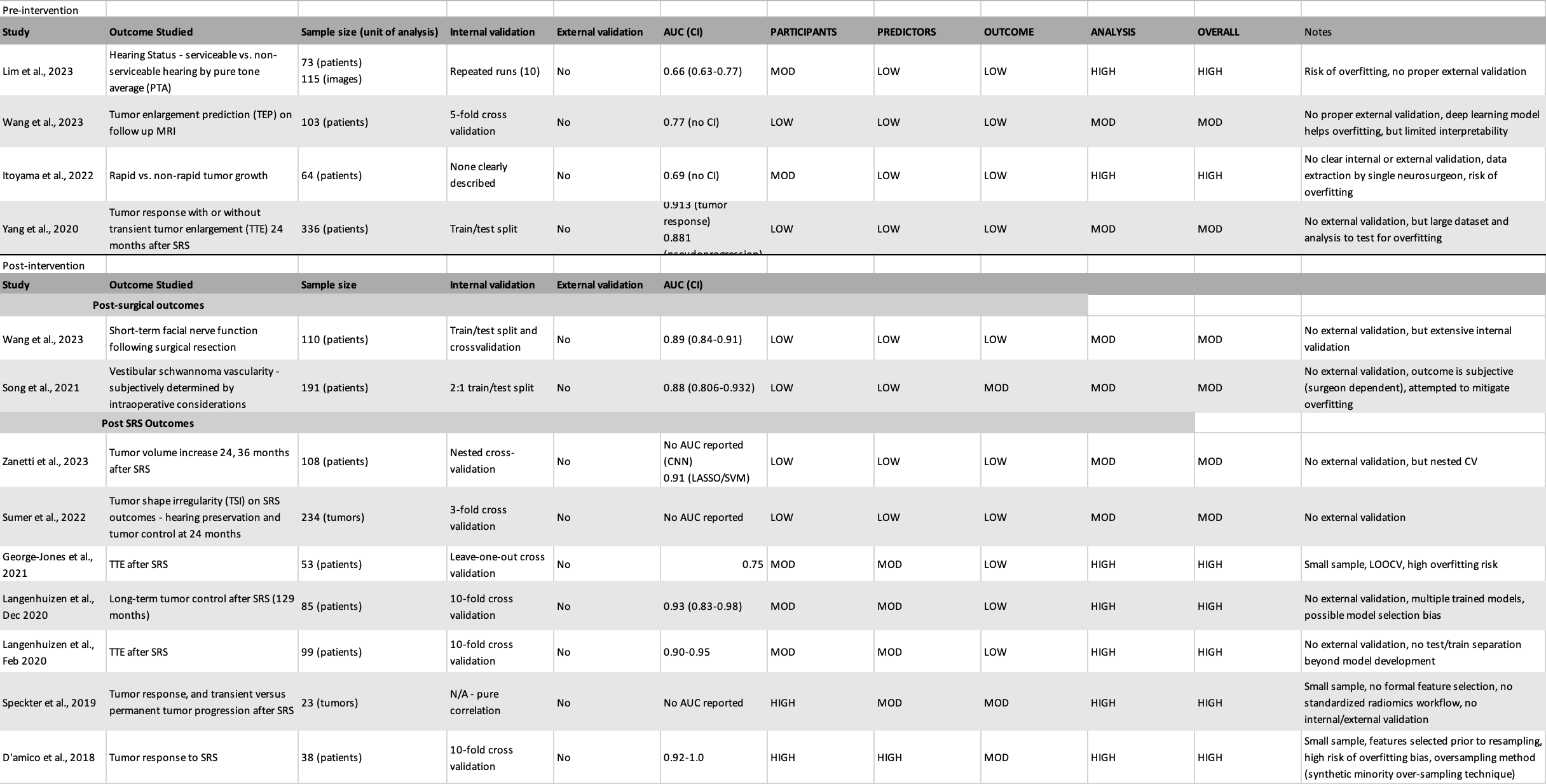

Supplement: Supplementary file 2 — Supplementary Material 2 [file 11060_2026_5663_MOESM2_ESM.docx]
